# Short-course radiotherapy followed by neo-adjuvant chemotherapy in locally advanced rectal cancer – the RAPIDO trial

**DOI:** 10.1186/1471-2407-13-279

**Published:** 2013-06-07

**Authors:** Per J Nilsson, Boudewijn van Etten, Geke AP Hospers, Lars Påhlman, Cornelis JH van de Velde, Regina GH Beets-Tan, Lennart Blomqvist, Jannet C Beukema, Ellen Kapiteijn, Corrie AM Marijnen, Iris D Nagtegaal, Theo Wiggers, Bengt Glimelius

**Affiliations:** 1 Department of Molecular Medicine and Surgery, Karolinska Institutet and Center for Surgical Gastroenterology, Karolinska University Hospital, Solna P9:03, SE 171 76 Stockholm, Sweden; 2 Department of Surgery, University Medical Center Groningen, Groningen, The Netherlands; 3 Department of Medical Oncology, University Medical Center Groningen, Groningen, The Netherlands; 4 Department of Surgical Science, Uppsala University, Uppsala, Sweden; 5 Department of Surgery, Leiden University Medical Center, Leiden, The Netherlands; 6 Department of Radiology, Maastricht University Medical Center, Maastricht, The Netherlands; 7 Department of Diagnostic Radiology, Karolinska University Hospital Solna, Stockholm, Sweden; 8 Department of Radiation Oncology, University Medical Center Groningen, Groningen, The Netherlands; 9 Department of Medical Oncology, Leiden University Medical Center, Leiden, The Netherlands; 10 Department of Clinical Oncology, Leiden University Medical Center, Leiden, The Netherlands; 11 Department of Pathology, Radboud University, Nijmegen Medical Center, Nijmegen, The Netherlands; 12 Department of Radiology, Oncology and Radiation Science, Uppsala University, Uppsala, Sweden; 13 Department of Oncology and Pathology, Karolinska Institutet, Solna, Sweden

**Keywords:** Rectal cancer, Radiotherapy, Chemotherapy, Neo-adjuvant, Magnetic resonance imaging

## Abstract

**Background:**

Current standard for most of the locally advanced rectal cancers is preoperative chemoradiotherapy, and, variably per institution, postoperative adjuvant chemotherapy. Short-course preoperative radiation with delayed surgery has been shown to induce tumour down-staging in both randomized and observational studies. The concept of neo-adjuvant chemotherapy has been proven successful in gastric cancer, hepatic metastases from colorectal cancer and is currently tested in primary colon cancer.

**Methods and design:**

Patients with rectal cancer with high risk features for local or systemic failure on magnetic resonance imaging are randomized to either a standard arm or an experimental arm. The standard arm consists of chemoradiation (1.8 Gy x 25 or 2 Gy x 25 with capecitabine) preoperatively, followed by selective postoperative adjuvant chemotherapy. Postoperative chemotherapy is optional and may be omitted by participating institutions. The experimental arm includes short-course radiotherapy (5 Gy x 5) followed by full-dose chemotherapy (capecitabine and oxaliplatin) in 6 cycles before surgery. In the experimental arm, no postoperative chemotherapy is prescribed. Surgery is performed according to TME principles in both study arms. The hypothesis is that short-course radiotherapy with neo-adjuvant chemotherapy increases disease-free and overall survival without compromising local control. Primary end-point is disease-free survival at 3 years. Secondary endpoints include overall survival, local control, toxicity profile, and treatment completion rate, rate of pathological complete response and microscopically radical resection, and quality of life.

**Discussion:**

Following the advances in rectal cancer management, increased focus on survival rather than only on local control is now justified. In an experimental arm, short-course radiotherapy is combined with full-dose chemotherapy preoperatively, an alternative that offers advantages compared to concomitant chemoradiotherapy with or without postoperative chemotherapy. In a multi-centre setting this regimen is compared to current standard with the aim of improving survival for patients with locally advanced rectal cancer.

**Trial registration:**

ClinicalTrials.gov NCT01558921

## Background

Over the past decades, management of rectal cancer has evolved immensely leading to improved patient outcomes. Although increased awareness, introduction of screening programmes [1] and enhanced perioperative care [2] have played roles in reducing mortality and morbidity rates, two distinct therapeutic developments have been of key importance. Firstly, surgical techniques have been refined [3,4] and disseminated to the colorectal surgical community [5]. Randomized controlled studies to prove the effects of new surgical techniques are difficult to design and run, but observational data clearly indicate benefits [6-8]. Secondly, (neo)adjuvant therapies including radiotherapy and chemotherapy have become integrated parts of rectal cancer management.

The Uppsala trial on pre- or postoperative radiotherapy for rectal cancer was the starting point for a line of development towards full acceptance for preoperative short-course radiotherapy [9]. The Stockholm I trial run in parallel [10], and the subsequent Swedish Rectal Cancer Trial with randomization between surgery alone and preoperative radiotherapy with 5 Gy x 5 in resectable rectal cancer showed not only improved local control but also a survival benefit [11]. Criticism against these trials regarding the quality of surgery performed was met in the Dutch TME trial where only surgeons trained in total mesorectal excision (TME) were operating. This study confirmed improved local control with the use of preoperative short-course radiotherapy although no significant effects on survival could be seen, apart from certain subgroups [12,13]. Randomized data indicating decreased short-term toxicity and no differences in long-term oncologic outcomes and late toxicity compared to preoperative chemoradiation in resectable rectal cancer suggest that short-course radiation should be preferred in these patients [14].

There is an overwhelming body of evidence supporting adjuvant chemotherapy in colon cancer with indisputable effects on survival [15]. However, in rectal cancer the corresponding evidence is weaker although a recent metaanalysis [16] showed favourable outcomes with the use of fluoropyrimidine-based chemotherapy. Another systematic overview questioned its use, particularly after preoperative chemoradiotherapy [17]. The risk of developing metachronous metastases in intermediate and locally advanced rectal cancer is 25–65% [18-20] and systemic chemotherapy aim to treat occult or micrometastatic disease that may later appear as distant metastases. At least two arguments against postoperative delivery of chemotherapy can be brought up. Firstly, rectal cancer surgery is connected with a substantial risk of postoperative complications that may lead to patient inability to tolerate postoperative chemotherapy [21,22]. Secondly, in an early disease phase when microscopic dissemination is limited the efficacy of systemic chemotherapy would theoretically be expected to be the greatest. Surgery, particularly if extensive, may also accelerate tumor growth [23,24]. Neoadjuvant chemotherapy has been proven favourable in the management of colorectal liver metastases [25], in gastric cancer [26,27] and is currently being studied in colon cancer in the ongoing FOxTROT trial [28].

Magnetic resonance imaging (MRI) is currently the most accepted modality for preoperative local staging of rectal cancer. Following the MERCURY-trial and further studies there are criteria, although not yet universally accepted or used, for identification of patients with a high risk of local and/or systemic relapse [29]. For patients with early, clearly resectable tumours (also designated “good”) surgery alone may provide excellent results with respect to both local control and survival. In slightly more advanced tumours, or “intermediate” following the terminology of the European Rectal Cancer Consensus Conference (EURECA-CC2) [30] or the European Society of Medical Oncology (ESMO) Consensus Guidelines [31], the increased risk of local recurrence justifies preoperative radiotherapy. Short-course radiotherapy with immediate surgery is a valid option in this situation as it reduces the risk of recurrence by 60-70% [32,33]. However, in this “intermediate” group (also designated “bad”), frequently termed “locally advanced”, chemoradiation is the preferred option by many [22,30,34]. In patients showing high-risk features on MRI, tumours best termed “locally advanced”, or “ugly”, there is a substantial risk of treatment failure either locally or systemically and chemoradiation is the reference regimen, since the addition of chemotherapy to conventionally fractionated radiotherapy improves local control and cancer-specific survival [35]. However, some patients may be considered too frail for chemoradiation and three recent reports have shown promising results with a strategy of delivering 5 Gy x 5 with delayed surgery [36-38]. These studies, although non-randomized, support the notion that also short-course preoperative radiation results in down-staging if surgery is postponed. In addition, a Dutch phase II trial [39], in which patients with resectable metastatic rectal cancer were given short-course radiation followed by preoperative chemotherapy including bevacizumab reported high response rates and radical (R0) resection was achieved in 80% of the patients.

Following evidence of tumour down-staging or down-sizing with short-course radiation and arguments for neo-adjuvant rather than adjuvant chemotherapy there is a rationale for applying this concept on patients with rectal cancer at high risk of local or systemic failure. The present RAPIDO (Radiotherapy And Preoperative Induction therapy followed by Dedicated Operation) trial has been designed to assess whether short-course radiation followed by up-front chemotherapy before surgery improves 3-year disease-free survival (DFS) in patients with locally advanced rectal cancer compared to conventional chemoradiation with optional (according to institution policy) postoperative adjuvant chemotherapy.

## Methods/design

### Study design

**Figure 1:**
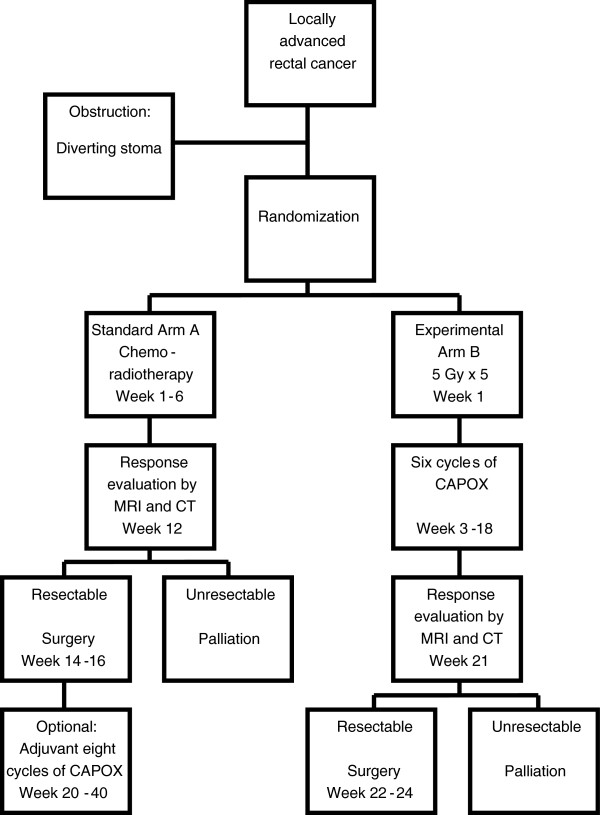
Treatment algorithm.

The study is a two-arm prospective randomized multicentre trial. The treatment algorithm is presented in Figure 1.

### Study objectives

The study compares two different preoperative regimens for locally advanced rectal cancer. The primary outcome measure is DFS after 3 years. Secondary objectives are to describe the toxicity profile, the rate of completion of preoperative treatment, the fraction of patients with a radical resection (R0) and to determine the rate of pathological complete response (pCR). Furthermore, local recurrence rate after 3 years follow-up, quality of life (QoL), functional outcome and overall survival (OS) are secondary endpoints.

### Trial organisation

The RAPIDO-trial has been jointly designed by Uppsala Akademiska Hospital, Sweden, Leiden University Medical Center, The Netherlands, University Medical Center Groningen, The Netherlands and Karolinska University Hospital, Stockholm, Sweden. The trial is investigator initiated and sponsors are the Dutch Colorectal Cancer Group (DCCG) and the Nordic Gastrointestinal Tumour Adjuvant Therapy Group (NGTATG). The RAPIDO-trial data management is financed by the Dutch and Swedish cancer societies.

### Coordination and monitoring

The trial is coordinated by the datacenter at Leiden University Medical Center, The Netherlands. The trial office is responsible for overall trial management, trial registration (ClinicalTrials.gov NCT01558921, EudraCT number 2010-023957-12, CKS number 2011–4997, NL36315.042.11, NTR 3230), database management and quality assurance. A monitoring committee appointed by the trial sponsors performs on-site monitoring of recruited patients according to the ICH Harmonized Tripartite Guideline for Good Clinical Practice (GCP).

### Ethics, informed consent and safety

The final protocol was first approved by the ethics committees of Uppsala University, Sweden and University Medical Center Groningen, The Netherlands. As centres have and will join this trial, appropriate approval from respective local ethics committee is obtained. This study is conducted in accordance with the most recent version of the Declaration of Helsinki and with the laws and regulations of each participating country. The protocol has been written, and the study will be conducted according to GCP. Written informed consent, signed and personally dated, is obtained from each patient before inclusion in the trial. Information that participation is voluntary and the nature, scope and possible consequences of the trial are explained to all patients by a physician. Prior to when valid consent has been obtained, the investigator will not undertake any measures specifically required only for the trial. Serious adverse events (SAE) or suspected unexpected serious adverse reactions (SUSAR), as defined by the protocol, will be reported to the datacenter at Leiden University Medical Center, The Netherlands and to the respective national trial coordinators who will report to competent authorities and ethical committees according to regulations applicable in each participating country.

### Statistics

DFS at 3 years is the primary end-point but the immediate anti-tumour secondary endpoints R0 and pCR rates are also of relevance. All efficacy analyses will be on an intention-to-treat basis. Per-protocol analyses will be performed as secondary analyses. Survival curves for DFS and OS will be plotted using the method of Kaplan and Meier. Cumulative incidence of local recurrence will be computed accounting for death as competing risk. Differences in survival will be tested with the log-rank test. Hazard ratios and 95% confidence intervals (CI) will be computed using Cox regression. All tests will be two-sided. A table will present the completion rate of the neo-adjuvant treatment, pCR frequency and percentages, fraction of patients with a R0 resection with 90 and 95% CI. Safety analyses will be based on treatment received and will include only eligible patients. Frequency and percentages for toxicity will be presented according to the Common Terminology Criteria for Adverse Events (CTCAE) version 4. All proportions will be presented with 95% CI. Fifty percent DFS is described in several studies with locally advanced rectal cancer patients [18-20,35]. The hypothesis is that the new treatment (Arm B) increases DFS after 3 years of follow-up from 50 to 60%. This difference corresponds to a hazard ratio of 0.737. A two-sided log rank test with a total of 452 DFS events achieves 90% power at α= 0.05 significance level to detect a hazard ratio of 0.737 when the proportion surviving in the control group is 50%. Based on four years of uniform accrual and two years of additional follow-up after the last patient has been included (six years total), a total of 842 evaluable patients will be required. With a drop-out of 5% the total number of patients to be included is 885. Interim analyses are planned with 50% and 75% of the DFS information for efficacy. Both interim analyses will be conducted by a team external to the sponsor team and each analysis will include the primary efficacy endpoint and key safety parameters.

### Randomization and stratification

Randomization is done centrally at the datacenter at Leiden University Medical Center, The Netherlands. Patients will be stratified according to institution, performance score (0 or 1), clinical T-stage (cT3 or cT4) and clinical node status (cN- or cN+).

### Patient selection

Inclusion in the RAPIDO trial requires that certain tumour and patient criteria are met. A staging MRI of specified quality is mandatory [40]. The inclusion and exclusion criteria are presented in Table 1. It is recognised that all MRI criteria indicate that the risk of systemic failure is high whereas all do not indicate an increased risk of local failure [29,41].

**Table 1:** Inclusion and exclusion criteria

**Inclusion criteria**	
General	Age ≥ 18 years
	ECOG performance score ≤ 1
	Written informed consent
	Staging done within 5 weeks prior to randomization
	Adequate potential for follow-up
	Mentally and physically fit for chemotherapy
	Adequate blood counts:
	White blood cell count ≥4.0 x 10^9^/L
	Platelet count ≥100 x 10^9^/L
	Clinically acceptable haemoglobin levels
	Creatinine levels indicating renal clearance ≥ 50 ml/min
	Bilirubin < 35 μmol/L
Primary tumour characteristics	Biopsy proven rectal adenocarcinoma*
	Locally advanced tumour fulfilling **at least one** of the following criteria on pelvic MRI:
	cT4a
	cT4b
	cN2
	Extramural vascular invasion (EMVI+)
	Involved mesorectal fascia (MRF+)
	Metastatic lateral lymphnodes (LN+)
**Exclusion criteria**	Extensive tumour growth into sacrum above S3
	Tumour involving lumbosacral nerve roots
	Distant metastasis (M1)
	Recurrent rectal cancer
	FAP or HNPCC
	Active Crohn’s disease or ulcerative colitis
	Concomitant malignancies (except basocellular carcinoma or in-situ cervical carcinoma)
	Known DPD deficiency
	Contraindications to MRI (e.g. pacemaker)
	Inability to give informed consent
	Concurrent uncontrolled medical condition
	Any investigational treatment for rectal cancer within past month
	Pregnancy or breast feeding
	Known malabsorption syndromes or lack of physical integrity of upper gastrointestinal tract
	Myocardial infarction within past 12 months or clinically significant cardiac disease
	Symptoms or history of peripheral neuropathy

*ECOG* Eastern Cooperative Oncology Group, * lower border of tumour < 16 cm with a rigid rectoscope, *MRI* Magnetic resonance imaging, cT4a, cT4b and cN2: Clinical stage according to TNM version 5, S3: sacral vertebra 3, *FAP* Familial adenomatous polyposis, *HNPCC* Hereditary non-polyposis colorectal cancer, *DPD* Dihydropyrimidine dehydrogenase.

### Preoperative therapy

Patients are randomized to one of two arms of preoperative treatments. Arm A is considered the standard arm and Arm B the experimental arm. In both arms, radiotherapy is delivered with CT-based 3D-conformal treatment planning with a defined pelvic clinical target volume (CTV). Arm A consists of chemoradiation with a dose to the planning target volume (PTV) of 45 Gy with 1.8 Gy in 25 fractions given 5 times a week. The dose to the boost PTV, encompassing the primary tumour and pathological lymph nodes is 5.4 Gy in 3 fractions leading to a total dose of 50.4 Gy. It is possible to deliver the treatment as 25 fractions of 2 Gy at centres who use this as their standard. An extra boost is possible to deliver towards the primary tumour area at risk of non-radical surgery [42] with 1.8 - 2 Gy x 2–4. The extra boost can be given intraoperatively (IORT). The radiotherapy is given in combination with capecitabin in a dose of 825 mg/m^2^ twice daily on all days of radiotherapy, including weekends. In Arm B, patients will receive a total dose of 25 Gy to the pelvic PTV delivered in fractions of 5 Gy during 5 days with a maximum overall treatment time of 8 days. An extra boost of 2 Gy x 2–3 is possible to deliver. With an optimal starting time at 11–18 days after the last day of radiotherapy, patients will receive neo-adjuvant chemotherapy. However, if radiation-related toxicity occurs, commencement of chemotherapy may be delayed up until 4 weeks after termination of radiotherapy. Chemotherapy is given in 3 week cycles and consists of capecitabine 1000 mg/m^2^ twice daily, day 1–14 combined with oxaliplatin 130 mg/m^2^ once every 3 weeks. In total, 6 cycles of chemotherapy are prescribed preoperatively. Treatment-related toxicity is monitored through the preoperative phase and dose modification can be made according to specified protocol schedules.

### Response and resectabilty evaluation

Patients randomized to Arm A will undergo response evaluation with MRI of the pelvis and computerized tomography (CT) of chest and abdomen after the full course of chemoradiation prior to surgery. Patients in Arm B who have received short-course radiotherapy and neoadjuvant chemotherapy will have MRI and CT after the last cycle of chemotherapy but prior to surgery. An additional response evaluation with MRI and CT is possible to perform after the third neo-adjuvant cycle. Response evaluation will be at a multidisciplinary team conference and patients with progressive or irresectable disease will receive palliative treatment. Radiological assessment will be according to the Response Evaluation Criteria in Solid Tumours (RECIST 1.1) [43].

### Surgery and histopathology

In Arm A surgery should be performed 6–8 weeks after termination of chemoradiation. In Arm B patients should undergo surgery 2–4 weeks after the last cycle of neoadjuvant chemotherapy. Surgical treatment should not differ between the two trial groups and can be done open or laparoscopically. Surgery should be performed according to the TME principles, however, in tumours located in the proximal part of rectum partial mesorectal excision (PME) is permitted provided that a 5 cm distal margin in mesorectum can be safely obtained. Surgery may include anterior resection, abdominoperineal resection or a low Hartmann’s procedure. Potentially invaded structures are resected *en bloc* with rectum. Pathological evaluation of resected specimens will be according to guidelines included in the study protocol. The 5^th^ edition of TNM will be used. In addition, circumferential resection margin (CRM) will be assessed and a margin of 1 mm or less is considered positive. CRM will be measured both for the primary tumour and for lymph nodes or tumour deposits, when present. Tumour regression grade (TRG) will be assessed according a three-tier grade (no regression, regression, and complete regression). Also, the quality of the resected specimen will be evaluated with separate scoring for the mesorectum and the anal canal. After inclusion of the last patient, a committee of experienced rectal pathologists will be appointed for central review of histopathology.

### Adjuvant chemotherapy and follow-up

Patients who receive chemoradiotherapy in Arm A can be treated with postoperative chemotherapy according to the local protocol of each participating centre. If patients are eligible for postoperative chemotherapy this should consist of 8 cycles of capecitabine 1000 mg/m^2^ twice daily, day 1–14 combined with oxaliplatin 130 mg/m^2^ once every 3 weeks. Patients treated in Arm B with short-course radiotherapy and neoadjuvant chemotherapy do not receive any postoperative chemotherapy. A minimum standardized follow-up schedule for all included patients is prescribed. This includes visits with history including morbidity/toxicity assessment, physical examination and measurement of carcinoembryonic antigen (CEA) at 6, 12, 36 and 60 months. Toxicity will be assessed and recorded according to CTCAEv4.0. QoL assessment using EORTC questionnaires QLQ-C30 and QLQ-CR29 will be performed at 36 and 60 months. All patients will also have CT of thorax and abdomen (or chest x-ray and liver ultrasonography) at 12 and 36 months. All suspicious findings should prompt further evaluation and examinations.

### Translational research

It is highly desirable to attach translational research to provide insights to prognosis and prediction of response to radiation and chemotherapy to the RAPIDO-trial. Analyses of both tumour tissue and serum/plasma with tissue microarray, proteomics and genomics would all generate increased knowledge. Hence, a schedule for collection of serum/plasma and of fresh tissue for freezing, at different stages of treatment in each arm, is defined in the study protocol. Pending on local resources and regulations, participation in the translational part of RAPIDO is optional.

## Discussion

Improved staging, introduction of multidisciplinary decision-making, refined surgery and appropriate use of preoperative radiotherapy, together with quality assessment by pathology and registries have all contributed to substantially lowering rates of local recurrence in rectal cancer from historical figures of above 30% to below 10% in many cohorts. Although there are certain subgroups who still suffer a high risk of not having R0 surgery or a local failure, the problem of local control can be seen as solved for a majority of rectal cancer patients. However, the improvements regarding local control achieved over the past decades are not matched by same-size improvements with respect to survival. Without compromising local therapy, it may therefore be justified to shift focus from local control to systemic control and survival when designing trials aiming at further development of rectal cancer treatment.

The primary aim of adjuvant systemic chemotherapy is to treat occult disease dissemination that may later occur as distant metastases. Current standard for locally advanced rectal cancer includes preoperative chemoradiation but, because of the risk of toxicity, dosage of chemotherapeutic agents must be reduced which may negatively affect the systemic efficacy. In many centres additional chemotherapy is administered postoperatively. However, since rectal cancer surgery is afflicted with high rates of postoperative complications, a substantial number of eligible patients are not fit to receive chemotherapy postoperatively [21,22,44]. In addition, when preoperative chemoradiation is administered (5 weeks) followed by surgery after 6–8 weeks and patients having to recover after surgery for 5–6 weeks, postoperative chemotherapy for suspected occult metastases cannot be delivered until after 4–5 months. On the other hand, neo-adjuvant chemotherapy yields favourable outcomes in oesophageal and gastric cancer [26,27], in colorectal hepatic metastases [25] and is currently investigated in primary colon cancer [28]. In contrast to these trials, in which chemotherapy is delivered pre- and postoperatively, all chemotherapy is given preoperatively in the experimental arm of the RAPIDO-trial. This was also the regimen in the Dutch “M1”-trial [39]. In this trial 50 patients with metastatic rectal cancer received short-course radiation followed by 6 cycles of full-dose capecitabine/oxaliplatin and bevacizumab preoperatively. The completion rate for all 6 cycles of chemotherapy was 85% and more than 90% received 4 cycles or more, and toxicity reported was mostly mild. Thus, the assumption that preoperatively administered chemotherapy is more likely to be accepted in full-dose than concomitant or postoperative appears to be valid.

Two randomized trials have compared short-course preoperative radiotherapy to preoperative chemoradiation in patients with resectable rectal cancer [45,46]. No evidence that chemoradiation was superior concerning local control or survival was provided in these studies. Furthermore, there were no indications of differences in late toxicity between the two regimens and short-course radiation resulted in less acute toxicity [14]. In these studies, in which patients underwent surgery the immediate week following short-course radiotherapy down-staging occurred to a much greater extent in patients who had received chemoradiation with delayed surgery. However, in an interim analysis of the on-going Stockholm III trial, also in resectable (intermediate stage) rectal cancer, the rate of pCR after short-course radiotherapy with delayed surgery was 12.5% [47]. Additionally, there are also other randomized data [39] and observational data [36-38] indicating a down-staging effect of short-course radiotherapy, given that surgery is after a delay. Among patients who underwent resection of the primary tumour in the “M1”-trial (approximately 75% with T3/T4N+ tumours), 91% had a R0 resection and pCR was found in 27% [39]. None of the included patients was considered inoperable because of primary tumour progression and only one patient suffered local symptoms which occurred due to massive tumour response with tumour necrosis and an abscess. The observational data from Stockholm also indicate that the risk of tumour progression during the “waiting time” is low even if chemotherapy is not administered in this period [38,47]. With this background, a rationale to test short-course radiation also for the locally advanced tumours appears to exist.

The RAPIDO-trial is designed with the aim of improving survival without compromising local control in patients with locally advanced rectal cancer. The aforementioned support in the literature for down-staging following short-course radiation with delayed surgery and the rationale for neo-adjuvant chemotherapy opens a window to test the combination of these two concepts, and to compare results with current standard being preoperative chemoradiotherapy with or without adjuvant chemotherapy. Although it is reasonable to assume that neo-adjuvant chemotherapy not only has systemic effects but also acts on the primary tumour it would be difficult to gain acceptance for a trial in which surgery is postponed after a prolonged chemotherapy period. Even if chemotherapy with the addition of biologics has improved substantially during the past decade [48], the medical therapy has limited cell kill effect and is the weakest component in the treatment armamentarium. The initial 5 Gy x 5 will prevent local progression during the prolonged chemotherapy aimed at killing all potential subclincal cancer cells, without postponing the start of the systemic therapy more than marginally. Most adjuvant schedules in colorectal cancer consist of 8 cycles (24 weeks) of chemotherapy but in the “M1”-trial only 6 cycles were given preoperatively [39], an approach that reduces the “waiting time” between short-course radiation and surgery. Bevacizumab was included in that trial but there is no evidence supporting an effect of either bevacizumab or cetuximab against sub-clinical disease [49,50] which renders it superfluous in the RAPIDO setting.

In the RAPIDO-trial, the logistically simple approach with initial local therapy with short-course radiotherapy followed by systemic adjuvant full-dose up-front chemotherapy in 6 cycles, before local treatment is finalized with surgery according to TME-principles, is being explored. If this concept in the RAPIDO trial yields improved survival with maintained results regarding local control, the established management of locally advanced rectal cancer with preoperative chemoradiation is challenged.

## Competing interests

The authors have no competing interests to declare.

## Authors’ contributions

The study protocol was originally conceived by BG, GAPH, LP and CJHvdV. The study protocol was drafted by BvE and PJN with the exception of parts relating to radiology which were drafted by LB and RGHB-T and parts relating to pathology which were drafted by IDN. JCB, EK, CAMM and TW have all participated in critical review relating study design and protocol. This article was conceived and drafted by PJN and BG. Critical review and contributions for finalising the article were provided by all other authors. All authors have read and approved both the study protocol and this article.

## Pre-publication history

The pre-publication history for this paper can be accessed here:

http://www.biomedcentral.com/1471-2407/13/279/prepub
